# Peritoneal carcinosis in male germ cell tumor patients: a registry study compiled by the German Testicular Cancer Study Group (GTCSG)

**DOI:** 10.1007/s00345-021-03905-0

**Published:** 2022-01-07

**Authors:** Christoph Seidel, Marcus Hentrich, Stefanie Zschäbitz, Pia Paffenholz, Axel Heidenreich, Tim Nestler, Ben Tran, Stefanie Fischer, Gedske Daugaard, Sebastian Ochsenreither, Margarida Brito, Friedemann Zengerling, Constantin Schwab, Carsten Bokemeyer, Christoph Oing

**Affiliations:** 1 Department of Oncology, Hematology and Bone Marrow Transplantation with Division of Pneumology, University Medical Center Hamburg-Eppendorf, Martinistraße 52, 20246 Hamburg, Germany; 2 Department of Medicine III-Haematology/Oncology, Red Cross Hospital, Munich, Germany; 3 Department of Medical Oncology, National Centre for Tumour Diseases, Heidelberg University Hospital, Heidelberg, Germany; 4 Department of Urology, University Hospital Cologne, Cologne, Germany; 5 Department of Urology, Federal Armed Services Hospital Koblenz, Koblenz, Germany; 6 Peter MacCallum Cancer Centre, Melbourne, Australia; 7 Department of Medical Oncology and Hematology, Cantonal Hospital St. Gallen, St. Gallen, Switzerland; 8 Department of Oncology, Copenhagen University Hospital, Rigshospitalet, Copenhagen, Denmark; 9 Department of Medical Oncology and Hematology, Charité Campus Benjamin Franklin, Berlin, Germany; 10 Charité Comprehensive Cancer Center, Charité Campus Mitte, Berlin, Germany; 11 Instituto Português de Oncologia de Lisboa, Lisboa, Portugal; 12 Department of Urology, University Hospital Ulm, Ulm, Germany; 13 Department of Pathology, National Centre for Tumour Diseases, Heidelberg University Hospital, Heidelberg, Germany; 14 Mildred Scheel Cancer Career Center HaTriCs4, University Cancer Center Hamburg, University Medical Center Hamburg-Eppendorf, Hamburg, Germany

**Keywords:** Testicular cancer, Germ cell tumor, Peritoneal carcinosis

## Abstract

**Purpose:**

To report on the clinical characteristics, outcome, and frequency of peritoneal carcinosis (PC) in patients with advanced germ cell tumors (GCT), a multicenter registry analysis was carried out.

**Methods:**

A multicenter registry analysis was conducted by the German Testicular Cancer Study Group (GTCSG) with international collaborators. Data was collected and analyzed retrospectively. Patients were eligible for inclusion if PC was diagnosed either by radiologic or histopathologic finding during the course of disease. Descriptive and explorative statistical analysis was carried out with cancer-specific survival (CSS) as primary study endpoint.

**Results:**

Collaborators from ten GCT expert centers identified 28 GCT (0.77%) patients with PC after screening approximately 3767 GCT patient files and one case was contributed from a cancer registry request. Patients were diagnosed from 1997 to 2019 at a median age of 37 years (interquartile range, 13). Two patients (7%) presented with stage I and 27 patients (93%) with synchronous metastatic disease at first diagnosis. The primary histology was seminoma in seven (27%) and non-seminoma in 21 patients (72%). PC was detected after a median of 15.3 months from primary diagnosis (range 0–177) and two consecutive treatment lines (range 0–5), respectively. The median CSS from the time of detection of PC was 10.5 months (95%Confidence Interval 0.47–1.30) associated with an overall 2-year CSS rate of 30%.

**Conclusion:**

PC represents a rare tumor manifestation in GCT patients and was primarily associated with the occurrence of advanced cisplatin-refractory disease conferring to a dismal prognosis.

## Introduction

Testicular germ cell tumors (GCTs) are the most common solid organ malignancy among young men aged between 15 and 35 years [1]. Due to an excellent sensitivity towards cisplatin-based chemotherapy as a part of multimodal treatment approaches, advanced GCTs represent a curable malignant disease associated with 5-year survival rates ranging from 50 up to 96% in advanced disease stages [2–4]. Metastatic dissemination commonly involves the retroperitoneal lymph nodes, lymph nodes of the mediastinum, and lungs. Other metastatic sites may include liver, bone and brain which are less common and associated with adverse outcomes [5–7]. Until now, peritoneal carcinosis (PC) in GCT patients was described by case reports and small case series only, revealing merely little data concerning the frequency, potential causes of development and impact on the patient’s outcome [8–12]. The largest series of cases published so far, comprised the course of disease of five GCT patients with PC from a French high-volume center. As four of the patients received retroperitoneal lymph node dissection (RPLND) prior to the detection of PC, the authors hypothesized that RPLND may have caused a route of tumor extension from a lymphatic leakage during surgery which promoted consecutive PC development [8]. This hypothesis was, furthermore, shared by two other case reports, presenting single patient cases with GCT and PC development after RPLND or lymph node biopsy [9, 10]. Another case report, however, depicted the course of disease of a heavily pre-treated patient who developed PC after multiple, partially inadequately dosed treatment lines without receiving prior RPLND. Here, Abe et al. concluded that multiple treatment regimens that were applied could have increased the aggressiveness of tumor biology, associated with development of a chemo-resistant phenotype resulting in the development of PC at late-stage disease [12].

To report on the clinical characteristics, outcome, and frequency of PC in GCT patients, a multicenter registry analysis was carried out. It was also the aim to investigate the potential correlation between the occurrence of PC and prior RPLND as a route of dissemination.

## Patients and methods

### Study population and inclusion criteria

This multicenter registry analysis was conducted by the German Testicular Cancer Study Group (GTCSG) in association with international collaborators. Clinical information was collected retrospectively via pseudonymized case report forms (CRFs) from the medical records. CRFs were subsequently centrally stored and assessed at the University Medical Center Hamburg–Eppendorf. A total of 29 cases from eleven participating centers were collected and eligible for analysis. GCT patients were eligible for the study, if PC was detected by histological or radiologic examination any time during the course of the disease. Only male GCT patients were considered for analysis.

The study was approved by the local ethics committee of the Chamber of Physicians Hamburg (File Number: PV7058).

### Statistical analysis

The objective of this multicenter registry study was to provide data concerning the clinical characteristics, outcome, and frequency of PC in GCT patients. It was also the aim to investigate the potential correlation between the occurrence of PC and prior RPLND as a route of dissemination. To find prognostic factors patient characteristics were correlated with the outcome.

The cancer-specific survival (CSS) defined as the time from detection of PC until to the date of death from GCT disease was considered as primary study endpoint. Patients lost to follow-up were censored at the date of last visit. Survival analysis was conducted using the method of Kaplan–Meier [13] and log-rank test was applied to compare survival rates. The following patient characteristics were tested as potential prognostic factors: seminoma vs. non-seminoma, late recurrence after first line systemic treatment vs. early recurrence after first line systemic treatment, gonadal disease vs. extragonadal disease, IGCCCG good vs. intermediate vs. poor, local treatment of PC vs. no local treatment of PC, and synchronous metastatic disease vs. metachronous metastatic disease. A two-sided *p* value < 0.05 was considered statistically significant. Statistical analyses were conducted using statistical package for the social science version 18 (SPSS). Due to the low patient number a multivariate regression model was not conducted.

## Results

### Patient characteristics

Eleven participating institutions identified a total of 29 GCT patients with PC. Patients were first diagnosed with GCT from 1997 to 2019 at a mean age of 37 years at first diagnosis (range 19–60 years).The primary histology at first diagnosis was pure seminoma in seven (27%) and non-seminoma in 22 (72%) patients, respectively. Two patients (7%) were first diagnosed with stage I disease according to UICC [14] and 27 patients (93%) presented with synchronous metastatic disease at the time of their diagnosis with stage II (*n* = 9) and III (*n* = 18) disease, respectively. Patients with synchronous metastatic disease were classified as poor prognosis in 16 (55%), intermediate prognosis in six (21%), and good prognosis in seven (24%) patients according to the IGCCCG criteria [2]. Patient characteristics are described in detail in Table 1.

**Table 1:** Patient characteristics

Characteristics	Absolute number of patients *n* = 29	%
Median age (years)	37	(range 18–60)
Histology		
Seminoma	7	24%
Non-Seminoma	22	76%
UICC stage at primary diagnosis		
UICC I	2	7%
UICC Stage II	9	31%
UICC Stage III	18	62%
IGCCCG classification at primary diagnosis of stage II/III disease		
Good	7	24%
Intermediate	6	21%
Poor	16	55%
Primary site of the tumor		
Gonadal	23	79%
Extragonadal retroperitoneal	6	21%
Median number of treatment lines	3	(range 1–7)
Salvage therapy performed	19	66%
High dose chemotherapy performed	13	45%
First line systemic treatment	28	97%
BEP	18	62%
VIP	4	14%
EP	3	10%
TIP	1	3%
VIC	1	3%
HD-VIP	1	3%
Second line systemic treatment	24	83%
HD-CE	8	28%
TIP	7	24%
GO	4	14%
Cisplatin or Carboplatin + Etoposid	2	3%
HD-VIP	2	7%
CGP	1	3%
Third line systemic treatment	13	45%
ACO	1	3%
TIP	1	3%
GO(P/I)	10	34%
EP	1	3%
HD-CE	1	3%
Resection of metastases after first line treatment		
RPLND	13	45%
Atypical lung resection	1	2%
Removal of peritoneal lesions	1	2%

*BEP* Bleomycin, Etoposide, Cisplatin; *EP* Etoposide, Cisplatin; *VIP* Etoposide, Ifosfamide, Cisplatin; *TIP* Paclitaxel, Ifosfamide, Cisplatin; *VIC* Vindesin, Ifosfamide, Carboplatin; *HD VIP* High dose Etoposide, Ifosfamide, Cisplatin; *GO* Gemcitabine, Oxaliplatin; *CGP* Carboplatin, Gemcitabine, Paclitaxel; *ACO* Adriamycin, Cyclophosphamide, Vincristin

### Course of disease and treatment

The patients of this cohort received a median of three different systemic treatment lines from the timepoint of first diagnosis (range 1–7). Recurrence after first line systemic treatment was reported in 27 patients (93%) and two patients (7%) died during or prior to first line systemic treatment due to tumor progression and pneumonia (7%). Of 28 patients treated with first line systemic treatment 24 patients (86%) received further salvage systemic therapies. The most frequently applied salvage regimen was high dose carboplatin with etoposide and autologous peripheral stem cell transplantation (PBSCT) administered in eight patients followed by paclitaxel, ifosfamide and cisplatin (TIP) administered in seven patients. Of thirteen patients receiving consecutive third line treatment, gemcitabine and oxaliplatin was administered to ten patients. Details concerning the different chemotherapy lines administered are reported in Table 1. Surgical procedures which included the resection of metastases of the peritoneum were carried out in seven patients during their course of disease (24%) Additional treatment with Hyperthermic Intra-Peritoneal Chemotherapy (HIPEC) was administered to five patients (17%).

### Frequency and detection of PC

**Fig. 1:**
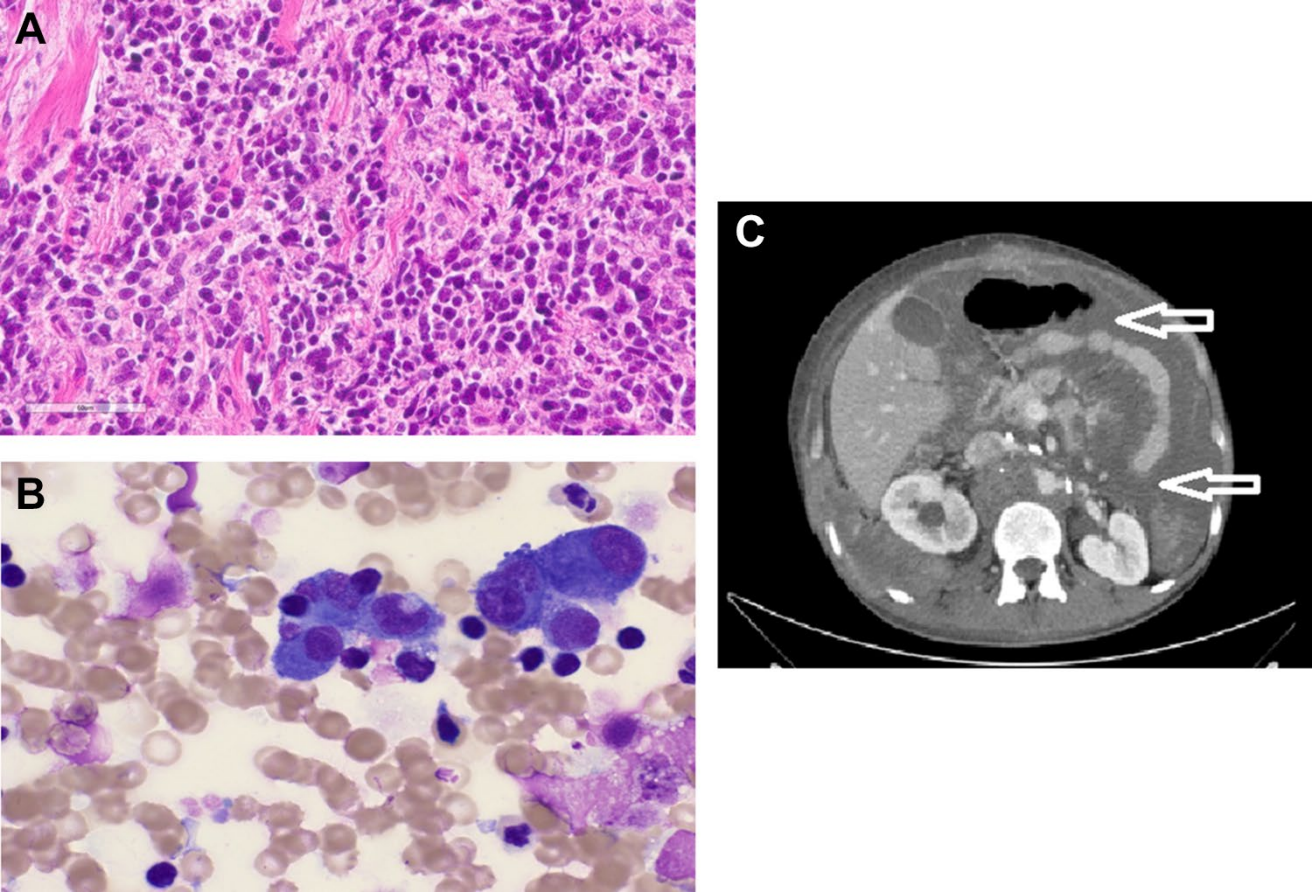
**A** Picture of immature teratoma, consisting of densely aggregated, nodular, atypical, small-to-medium-sized cell clusters (Coutesy Dr. Schwab, Heidelberg). **B** Cytological analysis of the ascitic fluid. May–Grünwald–Giemsa staining showed atypical pleomorphic cells with enlarged nuclei, dispersed chromatin and cytoplasmatic vacuoles, arranged as loosely cohesive clusters (Courtesy Dr. Heinz Diem, Munich). **C** CT scan schowing signs of peritoneal carcinosis (arrows) in a 26-year-old male with metastatic non-seminoma compromising intestinal loops leading to bowel obstruction

Altogether 28 cases were detected from ten centers after screening their databases which included approximately 3767 GCT patient files which corresponds to a prevalence of 0.74%. One additional case of PC was detected after the search from a local cancer registry. All participating centers were tertiary center hospitals and experienced in GCT patient care. Two participating institutions had exclusively access to patients with metastatic disease. PC diagnosis was confirmed with biopsy or surgical resection in six (29%) or by radiological examination in 23 cases (79%). Here histopathological evaluation revealed seminoma in three cases and choriocarcinoma, immature teratoma, and teratoma with adenocarcinoma and spindle cell malignant tumor in one case each. Results from the histological examination of the specimens if biopsy or surgical resections were carried out are depicted in detail in Table 1. Figure 1A illustrates an exemplar of the pathological examination of a resected PC lesion demonstrating immature teratoma and Fig. 1B displays carcinoma cells detected in ascites after paracenteses was carried out. Figure 1C shows the radiological finding of a patient from this cohort with PC lesions compromising intestinal loops leading to bowel obstruction.

### Development and symptoms of PC

PC was diagnosed after a median of 15.3 (95% CI 18.8–79.5) months from primary diagnosis and after a median of two consecutive lines of platinum-based chemotherapy, respectively (range 0–5). Clinical symptoms associated with PC were described in 15 of 29 cases (52%). In two patients (7%) data concerning the occurrence of symptoms was not specified by the investigators and concerning 12 patients (41%) no PC-specific symptoms were documented. The most frequent symptoms associated with PC were abdominal pain in seven (24%), ascites in five (17%) and ileus in three patients (10%). Further symptoms described are reported in Table 1. Paracenteses was carried out in six patients (21%) associated with a proof of malignant germ cell tumor cells by cytological examination in three patients (10%). Prior to the detection of PC, 13 patients (45%) received RPLND.

### Patient outcomes

**Fig. 2:**
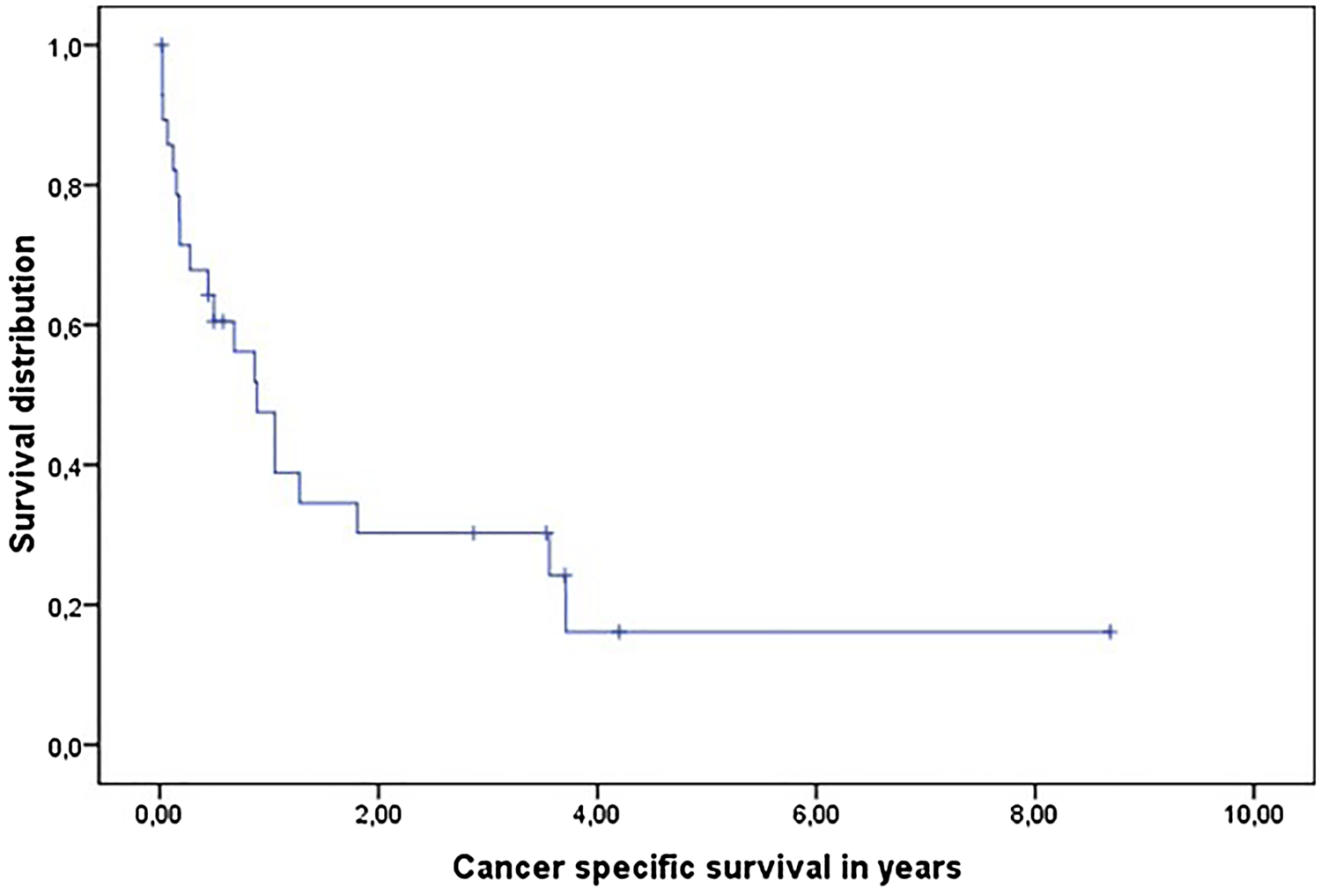
Cancer-specific survival in years from first diagnosis of peritoneal carcinosis

At the time of data acquisition nine patients (28%) were still alive and 20 patients (69%) had succumbed to their disease associated. The median CSS from PC diagnosis was 10.5 months (range 0.30–104.22) associated with a 2-year CSS rate of 30% (Fig. 2). Concerning patient characteristics with a potential impact on CSS univariate statistical analysis revealed no patient characteristics that significantly correlated with CSS (Table 2).

**Table 2:** Results of univariate analysis concerning CSS

Factor	2-year CSS rate	*p* value
Seminoma vs. Non-Seminoma	29% vs. 30%	0.807
Gonadal vs. Extragonadal	34% vs. 16%	0.704
Metachronous vs. Synchronous metastatic disease	100% vs. 25%	0.514
IGCCCG good vs. intermediate vs. poor	33% vs. 29% vs. 24%	0.692
Local PC treatment yes vs. no	22% vs. 16%	0.305

## Discussion

PC is a very rare condition in GCT patients and to date only single case reports and a small series of five patients addressing this condition are available. The aim of this study was to present sufficient data to adequately describe the patient characteristics, outcome, approximate frequency and potential causes concerning the route of dissemination of PC in GCT patients.

With a multicentric registry complied by the German Testicular Cancer Study Group (GTCSG) 28 GCT patients with PC were detected after screening approximately 3,767 patients, while one additional case was contributed from a regional cancer registry request. With a total of 29 cases this is so far the largest report on PC in GCT. With an approximate prevalence of 0.77%, our results confirm the observation by Andre et al. that the occurrence of PC from GCT is very rare but not exceptional. As earlier reports postulated a correlation between the development of PC and prior RPLND or lymph node biopsy [8–10] our analysis revealed that only 45% of patients received RPLND prior to the detection of PC. RPLND, therefore, seems not to be the only driver of PC development. In contrast, our cohort clearly depicts patients with adverse clinical characteristics, such as synchronous metastatic disease, poor prognosis according IGCCCG, and an exceptional high rate of cisplatin-refractory disease. Here 83% of our patients underwent at least two different treatment lines, while two patients died during or prior to first line systemic treatment. An overall 2-year CSS rate of 30% underlines this observation and is in line with previous reports on patients failing two or more treatment lines [15, 16]. We, therefore, hypothesize that PC may be in most of the cases the result of aggressive, multiply relapsed and thus treatment-refractory tumors, associated with dismal prognosis.

Despite the fact that this presented series is the largest analysis of PC in GCT patients, so far, major limitations include the retrospective study design and the still limited number of cases available highlighting the rarity of PC in GCT. Furthermore, only tertiary specialised GCT centers participated and two participating centres only had access to patient files with disseminated disease only which will probably lead to a higher incidence of PC.

In conclusion, peritoneal carcinosis is a rare phenomenon occurring in advanced and mostly heavily pretreated germ cell tumor patients who generally face a dismal prognosis.
